# Demystification of palliative care: what palliative care teams don’t want you to think about them

**DOI:** 10.1007/s12254-018-0420-2

**Published:** 2018-07-17

**Authors:** Eva K. Masel, Gudrun Kreye

**Affiliations:** 1 Clinical Division of Palliative Care, Department of Internal Medicine I, Medical University Vienna, Vienna, Austria; 2 Palliative Care Unit, Department of Internal Medicine II, University Hospital Krems, Karl Landsteiner University of Health Sciences, Krems an der Donau, Austria

**Keywords:** Caregivers, Death, Neoplasms, Palliative care, Quality of life

## Abstract

There is robust data that palliative care is prolonging life while there are still prejudices towards this discipline that have to be demystified. Patients, relatives and caregivers benefit from the concept of early integration of palliative care and therefore, palliative care should not be mainly regarded as end-of-life care.

The primary goal of palliative care is to consider the individual priorities and values of patients through specialized, interdisciplinary care and communication [1]. The “pallium” stands as a synonym for a coat in which the patients can slip into and that consists of individual, medical and psychosocial care, as well as spiritual care. This multimodal treatment should help patients and their relatives to gain a better understanding of the disease, its prognosis and possible treatment options. Palliative care should not be understood as a discontinuation of therapy, but more as a change in therapy goals [2]. Although there are many nonmalignant, chronic palliative disorders, the majority of patients receiving palliative care suffer from advanced oncological diseases.

There are still prejudices against the concept of palliative care that can be mitigated by well-published scientific studies. Here, we report upon some common myths towards palliative care.

## Myth 1: palliative care only deals with dying

By definition, palliative care should not be regarded as an alternative to a curative treatment and explicitly refers to treatment beyond that of only patients who are already in the final stages of an advanced disease. The aim of palliative care is to achieve prolongation of life with an improved quality of life. Such care should not be regarded just as an alternative to antineoplastic treatment; it can also be accompanied by antineoplastic therapy when such therapy is indicated [3]. The lives of patients with stage IV non-small-cell lung cancer was prolonged by three months compared to standard oncology treatment by initiation of early palliative care after diagnosis. The palliative care treatment consisted of at least a monthly visit by a palliative care team to assess physical and psychosocial impairments. Furthermore, the study results demonstrated that anxiety or depression were significantly lower in the group of patients receiving palliative care. Therefore, it can be concluded that palliative care does not lead to a loss of hope [4]. The results of this study could be underlined by further studies that confirmed prolongation of life through palliative care [5–7].

## Myth 2: palliative care mainly deals with talking

Sharing decision making, leading end-of-life discussions or initiating talks about the future are an elementary part of palliative care. However, palliation goes far beyond that and is an active form of therapy. Regardless of prognosis, its aim is to promote wellbeing throughout the course of a serious illness. This includes targeted improvement of symptoms as a major focus of palliative care teams [8]. Another domain of palliative care is to provide advance care planning [9]. Human beings tend to plan their whole lives through. In emergency situations, individual wishes often cannot be realized when advance care planning has not been provided in time.

## Myth 3: palliative care is demoralizing

It has been proven that end-of-life talks are held too late [10]. Medical staff should be encouraged to actively seek those conversations. The unspoken, so-called “elephant in the room” might turn “difficult” situations into “difficult” patients and families [11]. Most patients gratefully accept the offer for an end-of-life discussion, and the physician–patient relationship may be improved. A large number of patients want information about their prognoses [12]. Such conversations lead to a more realistic view of life expectancy on the part of patients without negatively affecting their emotional states or the physician–patient relationships [13].

So, when should palliative care begin? Experience has shown that patients are very appreciative of being actively informed about the options of palliative care teams and of their purposes [14]. Previous studies and research found that patients, relatives and caregivers benefit from the concept of early integration of palliative care for patients suffering from advanced cancer [15]. Why are there still barriers about offering palliative care to patients? Reasons are that palliative care is mainly regarded as end-of-life care, the fear that the patients would feel abandoned when offering palliative care to them or a concern that physicians would take away hope [16]. Another reason is a lack of resources. A high percentage of patients suffering from hemato-oncological diseases do receive antineoplastic therapies in the last month of life and die in intensive care units [17]. This represents the reality of a principle that can be described as “going down with waving flags” and which often prevents an examination of the fundamental question of the meaningfulness of an intervention. As part of a study with general practitioners, the assessment of the question “Would I be surprised if my patient dies within the next year?” revealed a high sensitivity concerning survival. Answering this question with a “No” would probably be the appropriate time to initiate advance care planning and information about the coming time. Whether people die in the hospital or at home also depends on socioeconomic factors. If information is provided early, home care and mobile palliative care teams can be organized on time. Palliative care units are still available for complex situations that cannot be handled at home. The level of early palliative care integration for oncology concepts still needs to be defined [18]. An issue that remains to be discussed is the feasibility of early integration due to lack of human resources in this field:

The organization and structure of a medical institution influences the way in which and at what time point palliative care is provided. Cooperation between different healthcare professionals is important to avoid parallel worlds where oncologists, palliative care specialists and mobile palliative care teams are separated from each other. In this case, patients, caregivers as well as colleagues might get the impression that they have to decide between the one or the other way. It seems to be a big fear of many patients that when the “palliative care door” opens, the “oncological door” is forever closed. In palliative care as well as in oncology there is no one-size-fits-all model. In terms of true interdisciplinarity it would be helpful to tell the patients that they do not lose their oncologist when palliative care is provided. Oncologists and palliative care specialists both should continue to communicate with the patients as well as with each other. In addition, there should be continuous training on the content and purpose of palliative care at all institutions.

Medical caregivers do practice a social profession. Therefore, interpersonal relationships have no clear guidelines and often require the ability to read between the lines. Linking this with evidence-based medicine is one of the strengths of palliative care. Palliative care is more than just prescribing opioids and sedatives. It gives attention to details and to the individual. Palliative care leads to a better quality of life, reduces the occurrence of anxiety and depression, can improve the burden of symptoms and unburdens caregivers. It is the approach of palliative care to look at the disease itself as well as to see the individual behind the disease.

## References

[1] Pandve HT, Fernandez K, Chawla PS, Singru SA (2009). Palliative care—need of awareness in general population. Indian J Palliat Care.

[2] Brennan F (2017). “To die with dignity”: an update on palliative care. Intern Med J.

[3] Kaasa S, Loge JH (2018). Early integration of palliative care—new evidence and old questions. Lancet.

[4] Temel JS, Greer JA, Muzikansky A, Gallagher ER, Admane S, Jackson VA (2010). Early palliative care for patients with metastatic non-small-cell lung cancer. N Engl J Med.

[5] Temel JS, Greer JA, El-Jawahri A, Pirl WF, Park ER, Jackson VA (2017). Effects of early integrated palliative care in patients with lung and GI cancer: a randomized clinical trial. J Clin Oncol.

[6] Greer JA, Jackson VA, Meier DE, Temel JS (2013). Early integration of palliative care services with standard oncology care for patients with advanced cancer. CA Cancer J. Clin..

[7] Bauman JR, Temel JS (2014). The integration of early palliative care with oncology care: the time has come for a new tradition. J. Natl. Compr. Canc. Netw..

[8] Masel EK, Berghoff AS, Schur S, Maehr B, Schrank B, Simanek R (2016). The PERS2ON score for systemic assessment of symptomatology in palliative care: a pilot study. Eur J Cancer Care (engl).

[9] Rietjens JAC, Sudore RL, Connolly M, van Delden JJ, Drickamer MA, Droger M (2017). Definition and recommendations for advance care planning: an international consensus supported by the European Association for Palliative Care. Lancet Oncol.

[10] Burki TK (2013). End-of-life discussions and care received. Lancet Oncol.

[11] Garner KK, Henager AA, Kirchner JE, Sullivan DH (2011). The elephant in the room: facilitating communication at the end of life. Fam Med.

[12] Oostendorp LJM, Ottevanger PB, van de Wouw AJ, Schoenaker IJH, de Graaf H, van der Graaf WTA (2014). Expected survival with and without second-line palliative chemotherapy: who wants to know?. Health. Expect..

[13] Enzinger AC, Zhang B, Schrag D, Prigerson HG (2015). Outcomes of prognostic disclosure: associations with prognostic understanding, distress, and relationship with physician among patients with advanced cancer. J. Clin. Oncol..

[14] Masel EK, Kitta A, Huber P, Rumpold T, Unseld M, Schur S (2016). What makes a good palliative care physician? A qualitative study about the patient’s expectations and needs when being admitted to a palliative care unit. PLoS ONE.

[15] Hui D, Kim YJ, Park JC, Zhang Y, Strasser F, Cherny N (2015). Integration of oncology and palliative care: a systematic review. Oncologist.

[16] LeBlanc TW, El-Jawahri A (2015). When and why should patients with hematologic malignancies see a palliative care specialist?. Hematology Am. Soc. Hematol. Educ. Program..

[17] Prigerson HG, Bao Y, Shah MA, Paulk ME, LeBlanc TW, Schneider BJ (2015). Chemotherapy use, performance status, and quality of life at the end of life. JAMA Oncol..

[18] Haun MW, Estel S, Rücker G, Friederich H-C, Villalobos M, Thomas M (2017). Early palliative care for adults with advanced cancer. Cochrane Database Syst Rev.

